# Thermo-responsive cell culture carriers based on poly(vinyl methyl ether)—the effect of biomolecular ligands to balance cell adhesion and stimulated detachment

**DOI:** 10.1088/1468-6996/16/4/045003

**Published:** 2015-07-28

**Authors:** Juliane Teichmann, Mirko Nitschke, Dagmar Pette, Monika Valtink, Stefan Gramm, Frauke V Härtel, Thomas Noll, Richard H W Funk, Katrin Engelmann, Carsten Werner

**Affiliations:** 1 Institute of Anatomy, Faculty of Medicine Carl Gustav Carus, Technische Universität Dresden, Fetscherstraße 74, D-01307 Dresden, Germany; 2 Leibniz Institute of Polymer Research Dresden, Max Bergmann Center of Biomaterials, Hohe Straße 6, D-01069 Dresden, Germany; 3 Herlac Coswig GmbH, Industriestraße 28, D-01640 Coswig, Germany; 4 Institute of Physiology, Faculty of Medicine Carl Gustav Carus, Technische Universität Dresden, Fetscherstraße 74, D-01307 Dresden, Germany; 5 CRTD/DFG-Center for Regenerative Therapies Dresden—Cluster of Excellence, Fetscherstraße 105, D-01307 Dresden, Germany; 6 Department of Ophthalmology, Klinikum Chemnitz gGmbH, Flemmingstraße 2, D-09116 Chemnitz, Germany

**Keywords:** thermo-responsive polymer, physico-chemical properties, biomolecular functionalization, poly(N-isopropylacrylamide), corneal endothelial cell sheet, tissue engineering, endothelial transplantation

## Abstract

Two established material systems for thermally stimulated detachment of adherent cells were combined in a cross-linked polymer blend to merge favorable properties. Through this approach poly(*N*-isopropylacrylamide) (PNiPAAm) with its superior switching characteristic was paired with a poly(vinyl methyl ether)-based composition that allows adjusting physico-chemical and biomolecular properties in a wide range. Beyond pure PNiPAAm, the proposed thermo-responsive coating provides thickness, stiffness and swelling behavior, as well as an apposite density of reactive sites for biomolecular functionalization, as effective tuning parameters to meet specific requirements of a particular cell type regarding initial adhesion and ease of detachment. To illustrate the strength of this approach, the novel cell culture carrier was applied to generate transplantable sheets of human corneal endothelial cells (HCEC). Sheets were grown, detached, and transferred onto planar targets. Cell morphology, viability and functionality were analyzed by immunocytochemistry and determination of transepithelial electrical resistance (TEER) before and after sheet detachment and transfer. HCEC layers showed regular morphology with appropriate TEER. Cells were positive for function-associated marker proteins ZO-1, Na^+^/K^+^-ATPase, and paxillin, and extracellular matrix proteins fibronectin, laminin and collagen type IV before and after transfer. Sheet detachment and transfer did not impair cell viability. Subsequently, a potential application in ophthalmology was demonstrated by transplantation onto de-endothelialized porcine corneas *in vitro*. The novel thermo-responsive cell culture carrier facilitates the generation and transfer of functional HCEC sheets. This paves the way to generate tissue engineered human corneal endothelium as an alternative transplant source for endothelial keratoplasty.

## 1. Introduction

Stimuli responsive polymers (SRP) [1] with a thermally stimulated volume phase transition like poly(*N*-isopropylacrylamide) (PNiPAAm) [2] or poly(vinyl methyl ether) (PVME) [3] can be prepared on solid surfaces by different techniques [4, 5]. When immersed in aqueous media, the abrupt change in solubility at the lower critical solution temperature leads to a reversible swelling and collapsing of the immobilized SRP coating upon small temperature changes. This effect was utilized for switchable cell culture carriers that allow for harvesting single cells or entire cell sheets without potentially harmful enzymatic treatments [6]. However, stimulated detachment of adherent cells including their extracellular matrix (ECM) [7] is a complex phenomenon that is strongly dependent on the cell type and the actual cultivation conditions. For a given SRP carrier, there may be cases where cell cultivation and detachment works as expected while other cells do not adhere properly or do not detach upon the same stimulus. To overcome this problem, several strategies were proposed to tune the physico-chemical properties of switchable cell culture carriers in order to meet cell type-specific requirements. This includes the immobilization of proteins or peptides to promote initial cell adhesion [8] as well as the inclusion of poly(ethylene glycol) units to weaken the overall cell–substrate interaction [9]. Furthermore, the thickness of a SRP coating was found to be an important parameter that affects cell adhesion and detachment [10]. The stiffness, the degree of swelling and the absolute switching amplitude of the SRP coating represent further potential tuning parameters [11].

In the study reported here, an attempt was made to merge the most favorable properties of two established material systems for stimulated cell detachment in a single SRP based cell culture carrier. Towards this goal the electron beam cross-linked blend of PVME and the alternating copolymer of vinyl methyl ether and maleic acid (PVMEMA), which was described previously by the authors [12], was complemented with an additional PNiPAAm component. This combines a wide parameter range for adjusting thickness, stiffness and swelling behavior as well as reactive sites for biomolecular functionalization with the outstanding interaction profile of PNiPAAm to improve cell detachment. The proposed thermo-responsive coating including a biomolecular functionalization is comprehensively characterized for a selected blend composition and an optimized set of preparation parameters. To illustrate the strength of this approach, the novel SRP-based cell culture carrier is applied to the generation of transplantable sheets of human corneal endothelial cells (HCEC).

The corneal endothelium represents the monolayer at the posterior part of the cornea and is formed by hexagonally shaped epithelial cells, which are called ‘corneal endothelial cells’ [13]. These cells are responsible for keeping the corneal hydration balance (‘deturgescence’) and thus maintain the corneal transparency [14]. HCEC show nearly no regenerative capacity *in vivo* [15]. Pathological cell loss caused by diseases (e.g. Fuchs’ endothelial dystrophy [16]) or by surgical or accidental traumata might therefore lead to a loss of function and consequently to opacification or even blinding of the affected eye. Approximately 2 million people suffer from corneal blindness worldwide [17]. Penetrating (full thickness [18]) or lamellar (partial [19]) corneal transplantation (keratoplasty) represent the common therapies. The lack of adequate corneal donor tissue with a sufficient endothelial cell density and quality prompted to the establishment of novel therapeutic approaches and techniques. Various tissue engineering strategies were developed during the last decades [20], including the generation of corneal endothelial cell sheets that were directly transplanted onto de-endothelialized corneas [21]. So far, these approaches were only experimental and did not reach clinical application in humans [22].

Results presented in this study were gained with the cell line HCEC-12 [23], subsequently indicated HCEC. This permanent cell line has a uniform morphology very similar to primary cells, which enables repetitive and comparative evaluation of the suitability of thermo-responsive carriers with different biofunctionalization for HCEC sheet generation. Contrary to other studies published so far, a two-step approach was followed: firstly, HCEC sheets were transferred onto planar, polymer-coated targets to work out the procedure without the geometrical challenges of concave corneal tissue. This intermediate step also allowed for a detailed analysis of cell morphology, vitality and functionality *before and after* detachment and transfer of HCEC sheets. Secondly, detached HCEC sheets were transplanted onto de-endothelialized porcine corneas *in vitro* to meet the geometrical challenges of the concave target tissue, and analyzed (immuno-)histochemically.

## 2. Materials and methods

### 2.1. Preparation of thermo-responsive cell culture carriers

A layer of 50%/40%/10% blend of PVME (TCI Europe, Zwijndrecht, Belgium), PNiPAAm (Sigma-Aldrich, Munich, Germany) and PVMEMA (Sigma-Aldrich) (PVME_50_–PNiPAAm_40_–PVMEMA_10_) was applied by spin coating on hydrophilized polystyrene thin films prepared on 20 mm microscope cover slips (Menzel Gläser, Braunschweig, Germany) or 15 × 20 mm^2^ silicon wafer precuts. Simultaneous cross-linking and immobilization was carried out with 150 keV electron beam irradiation (low energy electron facility ADU, advanced electron beams, Wilmington, USA) under nitrogen atmosphere at room temperature (RT) [5]. Samples were irradiated with an absorbed dose of 774 kGy applied stepwise to limit the temperature increase during electron beam treatment. Samples were rinsed in de-ionized (DI) water and ethanol to remove unbound material. Furthermore, porous membrane well inserts (Transwell^®^ transparent poly(ethylene terephthalate) (PET) membranes, pore diameter 0.4 *μ*m; Corning Life Sciences, Wiesbaden, Germany) were coated with PVME_50_–PNiPAAm_40_–PVMEMA_10_ with 100 *μ*l solution/well (0.5% in methanol) on a rocking shaker to obtain an even polymer coating by solvent evaporation. Simultaneous cross-linking and immobilization was carried out with 600 keV electron beam irradiation (electron beam device ELV-2, Budker Institute for Nuclear Physics, Novosibirsk, Russia) under nitrogen atmosphere at RT. Samples were irradiated with an absorbed dose of 774 kGy applied stepwise, and finally rinsed in DI water and ethanol.

### 2.2. Characterization of thermo-responsive cell culture carriers

The actual composition of the blend layer was determined by x-ray photoelectron spectroscopy (XPS; Amicus spectrometer, Kratos Analytical, Manchester, UK, accuracy for atomic composition +/−0.5 atomic percent (at%)). The swelling behavior of PVME_50_–PNiPAAm_40_–PVMEMA_10_ layers was characterized by spectroscopic ellipsometry (M-2000VI, J A Woollam, Lincoln, USA) on coated silicon wafers in a liquid media cell with phosphate buffered saline (PBS). A computer controlled heating device was used for temperature variation with a rate of 1 K min^−1^. Detailed information on preparation and characterization of SRP coatings is given in the supplementary information.

### 2.3. Biofunctionalization of thermo-responsive cell culture carriers

For covalent protein/peptide immobilization, the maleic acid groups of the PVMEMA blend component were converted into anhydride moieties by thermal annealing at 90 °C overnight [24]. Samples were cooled to RT and immediately incubated in a solution of either 10 *μ*g ml^−1^ laminin and 10 mg ml^−1^ chondroitin-6-sulfate (LN, CS, both Sigma-Aldrich; this particular combination of LN with CS was identified as an especially favorable coating for HCEC in previous studies [25]) or 5 *μ*g ml^−1^ cyclo(arginine–glycine–aspartic acid-D-tyrosine–lysine) (cRGD, Peptides International, Louisville, USA) in PBS for two hours at 37 °C under sterile conditions. As a negative control non-covalently (physisorptively) attached protein/peptide coatings were obtained likewise but without thermal annealing. Samples were subsequently exposed to growth medium F99_HCEC_, which consists of basal medium F99 (Ham’s F12 Nutrient Mixture/Medium 199; Biochrom AG, Berlin, Germany) supplemented with 5% fetal calf serum (Biochrom AG), 20 *μ*g ml^−1^ ascorbic acid (Sigma-Aldrich), 20 *μ*g ml^−1^ recombinant human insulin (Sigma-Aldrich), 10 ng ml^−1^ recombinant human basic fibroblast growth factor (Sigma-Aldrich) and antibiotics (2.5 *μ*g ml^−1^ amphotericin B and 50 *μ*g ml^−1^ gentamycin; Biochrom AG).

Protein displacement experiments were conducted as an analytical proof for durable protein attachment. For that purpose fluorescence-labeled Laminin-HiLyte 488 (Tebu-Bio, Offenbach, Germany) and fluorescein isothiocyanate-labeled RGD (RGD-FITC, kindly supplied by Dr Mikhail Tsurkan, Leibniz Institute of Polymer Research Dresden) were applied in the covalent versus non-covalent immobilization procedure. Subsequently, samples were exposed to serum-containing medium. Relative fluorescence intensities were measured with a fluorescence laser scanner (FLA-5100; FUJIFILM Europe GmbH, Düsseldorf, Germany) using an excitation wavelength of 473 nm to check for possible protein displacement, i.e., insufficient attachment of the biofunctional molecule.

### 2.4. Cell culture

The immortalized HCEC population HCEC-12 [23] was cultured in medium F99_HCEC_. Cells were subcultured by trypsinization and seeded at a density of 5 × 10^3^ cells cm^−2^ on T75 culture flasks (Corning Life Sciences) coated with 30 *μ*g LN and 30 mg CS in 3 ml PBS. HCEC were maintained at 37 °C in a humidified atmosphere containing 5% CO_2_. Medium was changed three times per week.

### 2.5. Cell adhesion

Thermo-responsive cell culture carriers were sterilized with 0.02% ProClin^®^300 Preservative (Sigma-Aldrich) in PBS at RT overnight, and washed with PBS to remove ProClin^®^300 residues. All following procedures were performed with pre-warmed (37 °C) PBS or F99_HCEC_ on a customized heating plate to prevent swelling of PVME_50_–PNiPAAm_40_–PVMEMA_10_ layers during sample handling and microscopy. Samples were incubated in F99_HCEC_ for 30 min before seeding of 5 × 10^4^ cells cm^−2^ or 8.5 × 10^4^ cells cm^−2^ onto cover slips or well inserts and cultured as described above. Adhesion, proliferation and monolayer formation were documented by repeated imaging over 8 days using phase contrast or Hoffman modulation contrast microscopy (Olympus IX50 by Olympus GmbH, Hamburg, Germany) with an AxioCam HR digital camera driven by AxioVision 4.7 software (Carl Zeiss MicroImaging GmbH, Göttingen, Germany).

### 2.6. Cell sheet detachment and transfer

Samples were handled on a customized heating plate at 37 °C to prevent unintended detachment of cell sheets. In the first experimental set, SRP coated cover slips with attached cell sheets were placed in 35 mm petri-dishes after eight days of culture and 10 *μ*l pre-warmed medium was added. A cellulose acetate membrane (18 mm diameter, VWR International GmbH, Darmstadt, Germany) was placed onto the HCEC layer, wetted with 20 *μ*l medium and the sample was incubated at 4 °C for 60 min to induce swelling of the SRP layer with consequential HCEC sheet detachment.

Detached HCEC sheets were carefully transferred with the membrane onto poly(octadecene-alt-maleic anhydride)-coated cover slips (see supplementary information for preparation), weighted down with sterile cover slips, and incubated with 90 *μ*l medium for at least four hours at 37 °C. Then, 3 ml of growth medium was added and the top cover slip and cellulose acetate membrane were removed. Transferred cell sheets were photo-documented by phase contrast or Hoffman Modulation contrast.

In the second set of experiments, HCEC sheets were detached as described above after eight days of culture. De-endothelialized corneas (see supplementary material for detailed information on the de-endothelialization procedure) were placed on silicone blocks with a concave center for corneal transplants (Geuder AG, Heidelberg, Germany). Sheets were transferred onto the de-endothelialized corneas with the cellulose acetate membranes centripetally cut 4x to facilitate adaption to the corneal curvature, and centrifuged at 100 g for 2 min to facilitate attachment of the cell sheet to the basement membrane of the corneal endothelium (Descemet’s membrane). Corneas were then placed endothelial side up in 12-well plates with 500 *μ*l F99_HCEC_ w/7.5% hydroxyethyl starch 130/0.4 (SUPRAMOL Parenteral Colloids GmbH, Rosbach vor der Höhe, Germany) per well at the epithelial and 100 *μ*l medium at the endothelial side. After at least four hours, 2.5 ml medium was added and the cellulose acetate membrane was removed.

In some cases, the cellulose acetate membrane was omitted to document detachment by time-lapse phase contrast microscopy.

### 2.7. Cell layer function

Formation of a closed monolayer was determined by measuring transepithelial electrical resistance (TEER, *Ω*cm^2^) with the impedance spectroscope cellZscope (nanoAnalytics, Münster, Germany) [26] of HCEC layers grown on PVME_50_–PNiPAAm_40_–PVMEMA_10_-coated well inserts biofunctionalized with LN/CS. The apical compartment corresponded to the anterior chamber and the basal compartment to the corneal stroma. Confluent cultures were subjected to a continuous TEER measurement over 48 h with a low alternating current of 3–100 kHz [27]. The background signal was determined with cell-free PET membrane well inserts with and without PVME_50_–PNiPAAm_40_–PVMEMA_10_-coating. Measurements were carried out at 37 °C in a humidified atmosphere containing 5% CO_2_ to prevent swelling of the SRP layer and consequential HCEC detachment.

### 2.8. Cell viability assay

In preliminary tests, cell viability was analyzed with YO-PRO^®^-1 (Life Technologies, Darmstadt, Germany) and propidium iodide (PI, Sigma-Aldrich;) to discriminate apoptotic cells (YO-PRO^®^-1-positive) and necrotic cells (YO-PRO^®^-1 and PI-positive) from vital cells (unstained) at one day after HCEC sheet transfer onto cover slips. Transferred cells were rinsed with PBS and incubated for 25 min at 37 °C in staining solution (1:1:48 composition of YO-PRO^®^-1 and PI in PBS w/1% serum). Further analyses were performed by vital staining with fluorescein diacetate (FDA, Sigma-Aldrich) and PI to discriminate between vital and necrotic HCEC. Transferred cell sheets were rinsed in PBS and incubated for 2 min at 37 °C in staining solution (1:32 000 FDA and 1:150 PI in PBS). Immediately after staining, samples were rinsed with 1% serum in PBS and photo-documented under a fluorescence microscope (Leica DM IRE2, Leica Microsystems) with Openlab 4.0.4 image processing software (Perkin Elmer, Improvision, Rodgau, Germany).

HCEC were harvested by trypsinization one day after transfer onto cover slips and the cells of four samples were pooled, pelleted, resuspended and analyzed by vital staining with 1 *μ*g ml^−1^ PI using a MACSQuant Analyzer (Miltenyi Biotec, Bergisch Gladbach, Germany). At least 10 000 cells were recorded for each analysis and calculations were performed with FlowJo software (Tree Star, Celeza GmbH, Olten, Switzerland).

### 2.9. Immunocytochemical staining

Protein production in HCEC grown for eight days on tissue culture polystyrene (TCP) or on biofunctionalized PVME_50_–PNiPAAm_40_–PVMEMA_10_ carriers was analyzed by immunocytochemical staining before and 24 h after transfer onto cover slips. Samples were stained for paxillin (cellular adhesion), fibronectin, collagen type IV and LN (ECM constituents), ZO-1 (tight junctions) and Na^+^/K^+^-ATPase *α*1 (ion pump).

Samples were rinsed with pre-warmed PBS (37 °C), fixed in 4% paraformaldehyde (Sigma-Aldrich) at 37 °C for 15 min and permeabilized with 0.5% Triton X-100 (Sigma-Aldrich) at RT for 10 min followed by nuclear staining with 2 *μ*g ml^−1^ Hoechst 33342 (Life Technologies) for 10 min. Samples were blocked in 10% goat or donkey serum (Dianova GmbH, Hamburg, Germany) in PBS for 30 min, followed by incubation with the primary antibodies diluted in 1% bovine serum albumin (BSA, Sigma-Aldrich) at RT (table 1). Samples were again blocked for 30 min and incubated with respective secondary antibodies (table 1) and Alexa Fluor^®^633 phalloidin (1:50; Life Technologies) in 1% BSA in PBS at RT in the dark for 45 min. Samples were rinsed with PBS, mounted on object slides with anti-fading mounting medium (O. Kindler GmbH, Freiburg, Germany), and photo-documented by confocal laser scanning microscopy with a Leica TCS SP5 and either a UV-diode (405 nm), argon laser (488 nm) or helium–neon laser (633 nm) (Leica Microsystems GmbH, Wetzlar, Germany) using Leica LAS AF image processing software.

**Table 1.** Primary and secondary antibodies; ICC—immunocytochemistry, IHC—immunohistochemistry.

Antigen	Host	Clone	Supplier	Dilution; incubation time
Paxillin	Mouse	349/paxillin	BD Transduction Laboratories, Heidelberg, Germany	1:100; 60 min
Fibronectin	Rabbit	Polyclonal	Biomol, Rockland Immunochemicals, Hamburg, Germany	1:200; 60 min
Collagen Type IV	Goat	Polyclonal	Biozol, Southern Biotech, Eching, Germany	1:20; overnight
Laminin	Rabbit	Polyclonal	Sigma-Aldrich, Munich Germany	1:20; overnight
ZO-1	Mouse	1/ZO-1	BD Transduction Laboratories, Heidelberg, Germany	ICC: 1:100; 60 min; IHC: 1:400; overnight
Na^+^/K^+^-ATPase *α*1	Mouse	464.6	Abcam, Cambridge, UK	ICC: 1:10; overnight; IHC: 1:400; overnight
Alexa Fluor^®^488 anti-mouse IgG	Goat	Polyclonal	Life Technologies, Molecular Probes, Darmstadt, Germany	1:200; 45 min
Alexa Fluor^®^488 anti-rabbit IgG	Goat	Polyclonal	Life Technologies, Molecular Probes, Darmstadt, Germany	1:200; 45 min
Alexa Fluor^®^488 anti-goat IgG	Donkey	Polyclonal	Life Technologies, Molecular Probes, Darmstadt, Germany	1:200; 45 min

Ellipsoid-shaped paxillin-associated focal adhesions were counted using the image processing software ImageJ (tool ‘cell counter’) and fluorescence intensity of samples stained for Na^+^/K^+^-ATPase *α*1 was quantified using the ImageJ tool ‘measurement of mean gray value’ [28, 29].

### 2.10. (Immuno-)histochemical analysis of corneal tissue

One day after transfer onto de-endothelialized porcine corneas, HCEC sheets were analyzed by histochemical and immunohistochemical staining. Porcine corneas with or without their own corneal endothelium served as controls. Corneas were fixed overnight at 4 °C with 4% formaldehyde in PBS, rinsed 2x for 60 min in 0.1 mol l^−1^ Sörensen buffer at RT and dehydrated in ascending ethanol concentrations to xylene. After embedding in paraffin, 5 *μ*m sections were cut on a rotating microtome (Reichert-Jung 2035, Leica Microsystems) and de-paraffinized by descending ethanol concentrations to distilled water. Histochemistry: samples were stained with hematoxylin and eosin (Sigma-Aldrich), dehydrated as described above, mounted in DePex (Serva Electrophoresis GmbH, Heidelberg, Germany), and photo-documented by light microscopy (OPTIPHOS-2, Nikon, Düsseldorf, Germany). Immunohistochemistry: after blocking with 10% goat serum in PBS for 30 min, samples were stained with primary antibodies (table 1) diluted in PBS and incubated at 4 °C overnight. Samples were washed, blocked again and incubated with the secondary antibody as described above. Slides were mounted with anti-fading mounting medium and staining was visualized and photo-documented under a BX 60 fluorescence microscope (Olympus Deutschland GmbH, Hamburg, Germany) equipped with a F-View CCD camera run by analySIS imaging software (Soft Imaging System GmbH, Münster, Germany).

### 2.11. Statistical analysis

Experiments on cell viability and function were performed at least in triplicates and data are presented as mean ± standard deviation (sd), unless otherwise stated. Unpaired Student's t-tests were performed and statistical significance was accepted at *p* < 0.05.

## 3. Results

### 3.1. Physico-chemical characterization of the SRP

**Figure 1.**
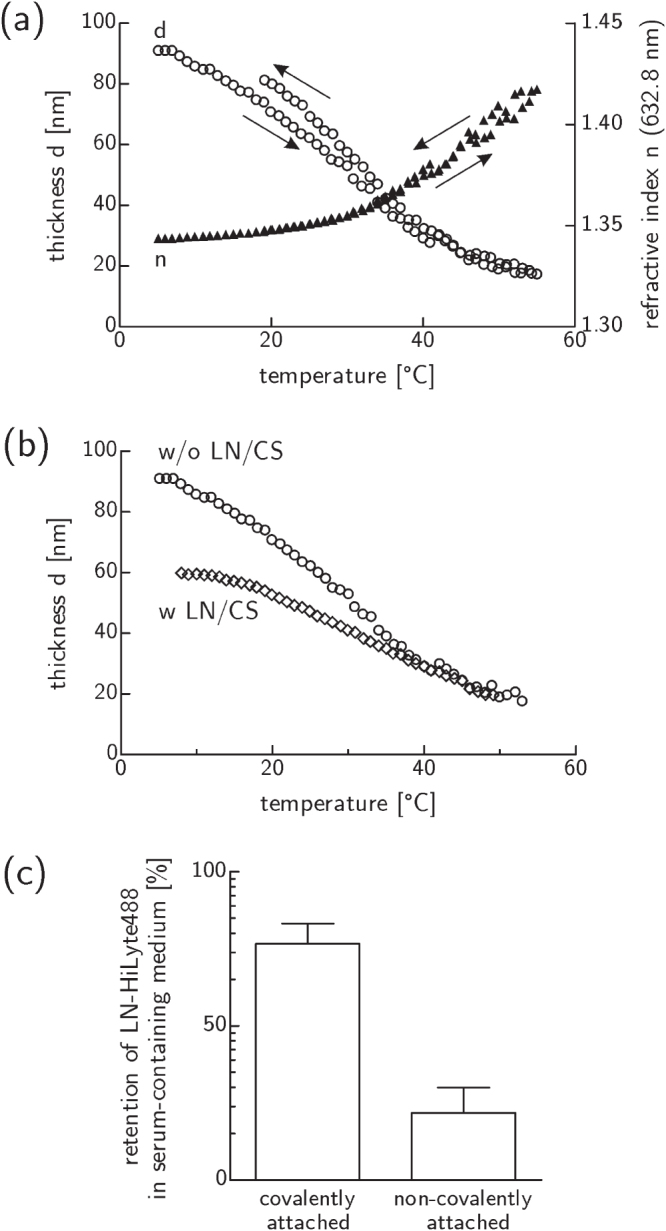
Switching and protein binding capabilities of PVME_50_–PNiPAAm_40_–PVMEMA_10_ layers. (a) Thermo-responsive switching in PBS was reversible with almost no hysteresis. (b) LN/CS binding moderately affected the switching behavior in PBS. (c) Exposure to serum containing medium at 37 °C proved the stability of covalently versus non-covalently attached LN. Mean ± sd of relative fluorescence intensity values; *n* = 2 (see figure S2 for corresponding RGD data).

Spectroscopic ellipsometry and XPS measurements revealed a dry thickness of about 20 nm for PVME_50_–PNiPAAm_40_–PVMEMA_10_ layers after simultaneous cross-linking and immobilization with an actual SRP layer composition as expected. PNiPAAm was properly integrated into the cross-linked blend with an absorbed dose of 774 kGy (also see table S1). *In situ* monitoring by spectroscopic ellipsometry revealed reversible swelling with a fully collapsed state above 50 °C and an expanded state at 20 °C with almost no hysteresis (figure 1(a)). The temperature dependencies of thickness and refractive index of hydrated SRP layers were consistent. Compared to pure PVME [5] the swelling characteristic of the blend is less distinct which can be attributed to the cross-linking of blend components with slightly different transition temperatures.

The anchorage of functional proteins/peptides on the SRP surface altered the thermo-responsive switching characteristic moderately as demonstrated for the binding of LN/CS (figure 1(b)). However, the system still responds properly to the thermal stimulus with a reduced amplitude of 40 nm instead of 70 nm w/o LN/CS. This can be explained as damping of the thermo-responsive behavior due to the addition of non-responsive material. The magnitude of the effect suggests a near monolayer coverage.

Fluorescently labeled ligands were immobilized properly only when the reactive anhydride sites had been activated during the preparation process, i.e., when a covalent binding mechanism was available (figures 1(c) and S2).

### 3.2. Cellular characterization of HCEC on thermo-responsive carriers

In preliminary studies thermo-responsive coatings with various dry film thicknesses of a few ten nm (‘thin’) versus a few hundred nm (‘thick’), cross-linking degrees of 258 kGy versus 774 kGy, and blend compositions were tested. It was observed, that a thin film 50%/40%/10% w/w blend of PVME, PNiPAAm and PVMEMA (PVME_50_–PNiPAAm_40_–PVMEMA_10_) supported cell adhesion, formation of a confluent cell layer and, finally, the stimulated detachment of the cell layer in a very good way. Detailed information on these screening experiments is given in the supplementary information and in figure S1. Therefore all following experiments were performed with PVME_50_–PNiPAAm_40_–PVMEMA_10_.

**Figure 2.**
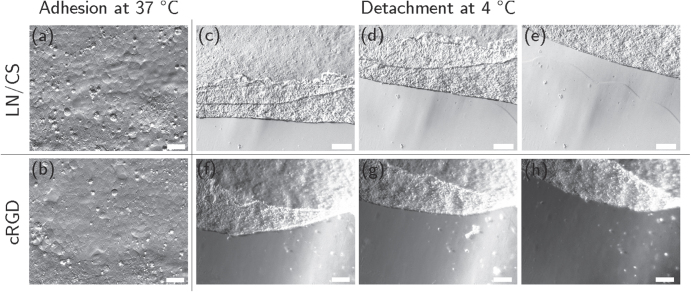
HCEC after eight days of culture on LN/CS- and cRGD-functionalized PVME_50_–PNiPAAm_40_–PVMEMA_10_ carriers. (a), (b) HCEC adhered and formed confluent monolayers of mostly polygonal cells at 37 °C (scale bar 50 *μ*m). (c)–(h) Representative frames from video sequences (see supplemental videos S1 and S2) to illustrate detachment of HCEC as a sheet upon temperature reduction (scale bar 200 *μ*m). *n* = 20.

**Figure 3.**
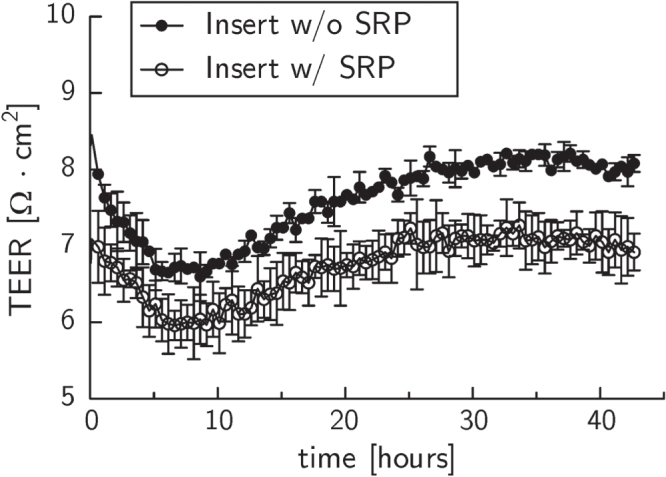
Transepithelial electrical resistance (TEER) measurements of confluent HCEC cultures on PET membranes without (closed circles) and with (open circles) coating of PVME_50_–PNiPAAm_40_–PVMEMA_10_. The difference between samples with or without SRP coating was statistically significant (*p* < 0.05) within each experiment. Displayed are averaged TEER values and the corresponding standard error of the mean of four samples per membrane type from a typical experiment. *p* = 0.0167.

After eight days of culture, HCEC formed a confluent monolayer of mostly polygonal cells on LN/CS- or cRGD-functionalized PVME_50_–PNiPAAm_40_–PVMEMA_10_ carriers (figures 2(a), (b) and S3; comparison to cells cultured on TCP see figures S4(a), (b)). TEER was slightly lower in HCEC sheets cultured on PVME_50_–PNiPAAm_40_–PVMEMA_10_ carriers (figure 3(c)) compared to controls. The TEER settled after approximately 24 h to 8 *Ω* cm^2^ for samples without PVME_50_–PNiPAAm_40_–PVMEMA_10_ and to 7 *Ω* cm^2^ for samples with PVME_50_–PNiPAAm_40_–PVMEMA_10_, respectively. The difference between samples with or without SRP coating was statistically significant (*p* < 0.05) within each experiment. A complete and fast detachment of HCEC as a sheet was achieved by incubating the samples at 4 °C for 60 min While LN/CS-functionalized PVME_50_–PNiPAAm_40_–PVMEMA_10_ carriers allowed for a good cell adhesion and also for faster and easier detachment, the adhesion promoting cRGD-functionalized carriers impaired HCEC sheet detachment, indicating the need for balancing adhesion and detachment (figures 2(c)–(h); videos S1 and S2; comparison to cells cultured on TCP see figures S4(c), (d)).

### 3.3. Molecular characterization of HCEC on thermo-responsive carriers

**Figure 4.**
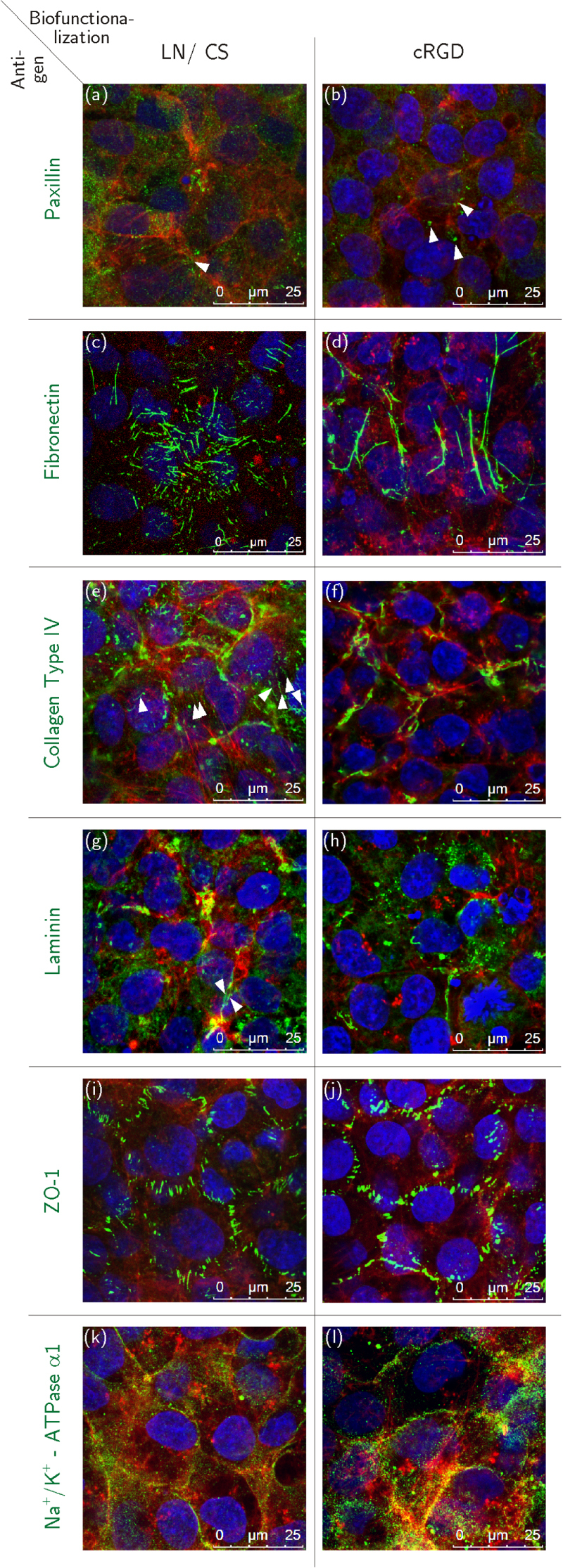
Immunocytochemical staining of HCEC before detachment; HCEC were cultured for eight days on PVME_50_–PNiPAAm_40_–PVMEMA_10_ carriers functionalized with LN/CS or cRGD. (a), (b) Dot-shaped paxillin signals (white arrowheads). (c), (d) Fibronectin fibers. (e), (f) Fine collagen type IV fibers (white arrowheads) and aggregated collagen type IV. (g), (h) Few fine laminin fibers (white arrowheads) and mostly aggregated laminin signals. (i), (j) ZO-1 localized to the lateral cell membranes. (k), (l) Strong Na^+^/K^+^-ATPase *α*1 signals associated to the lateral cell membranes. Antigens of interest are shown in green (Alexa Fluor^®^488), F-actin fibers in red (Phalloidin) and the nuclei in blue (Hoechst). *n* = 3.

Paxillin signals appeared predominantly dot-shaped in HCEC sheets on PVME_50_–PNiPAAm_40_–PVMEMA_10_ carriers that were biofunctionalized with either cRGD or LN/CS (figures 4(a) and (b)), while in HCEC grown on cRGD-functionalized TCP more ellipsoid paxillin signals were detected than on LN/CS-functionalized TCP samples (figures S5(a), (b) and S6). Similar to samples on TCP, fibronectin was deposited rather randomly in cell layers established on LN/CS-functionalized PVME_50_–PNiPAAm_40_–PVMEMA_10_ carriers, whereas fewer but longer fibronectin fibers were observed on cRGD-functionalized samples (figures 4(c), (d) and figures S5(c), (d)). Collagen Type IV appeared aggregated in all HCEC sheets irrespective of carrier material and biofunctionalization (figures 4(e), (f) and figures S5(e), (f)), except for LN/CS-functionalized SRP samples where some fine fibers were seen (white arrowheads in figure 4(e)). LN appeared aggregated in all HCEC sheets irrespective of carrier material and biofunctionalization (figures 4(g), (h) and figures S5(g), (h)), except for some fine fibers in HCEC cultured on LN/CS-functionalized TCP samples (white arrowheads in figures 4(g) and S5(g)).

Zonula occludens-1 (ZO-1) was localized at the lateral cell membranes in HCEC on LN/CS- and cRGD-functionalized PVME_50_–PNiPAAm_40_–PVMEMA_10_ and TCP carriers (figures 4(i), (j); figures S5(i), (j)). Strong signals for Na^+^/K^+^-ATPase *α*1 were detected at the lateral cell membranes of HCEC grown on cRGD-functionalized PVME_50_–PNiPAAm_40_–PVMEMA_10_ carriers (figure 4(l)) and TCP controls (figure S5(l)), while weaker signals were seen on LN/CS-functionalized SRP (figure 4(k)) and on TCP samples (figure S5(k)). Additionally the relative fluorescence intensities of the Na^+^/K^+^-ATPase *α*1 signals (figure S7) have been compared. A stronger relative fluorescence intensity was determined for HCEC grown on cRGD-functionalized PVME_50_–PNiPAAm_40_–PVMEMA_10_ carriers (figure S7 (b)) and TCP controls (figure S7 (d)), while fluorescence intensities were lower on LN/CS-functionalized SRP (figure S7 (a)) and TCP samples (figure S7 (c)).

### 3.4. Cellular characterization of HCEC transferred from thermo-responsive carriers onto planar targets

**Figure 5.**
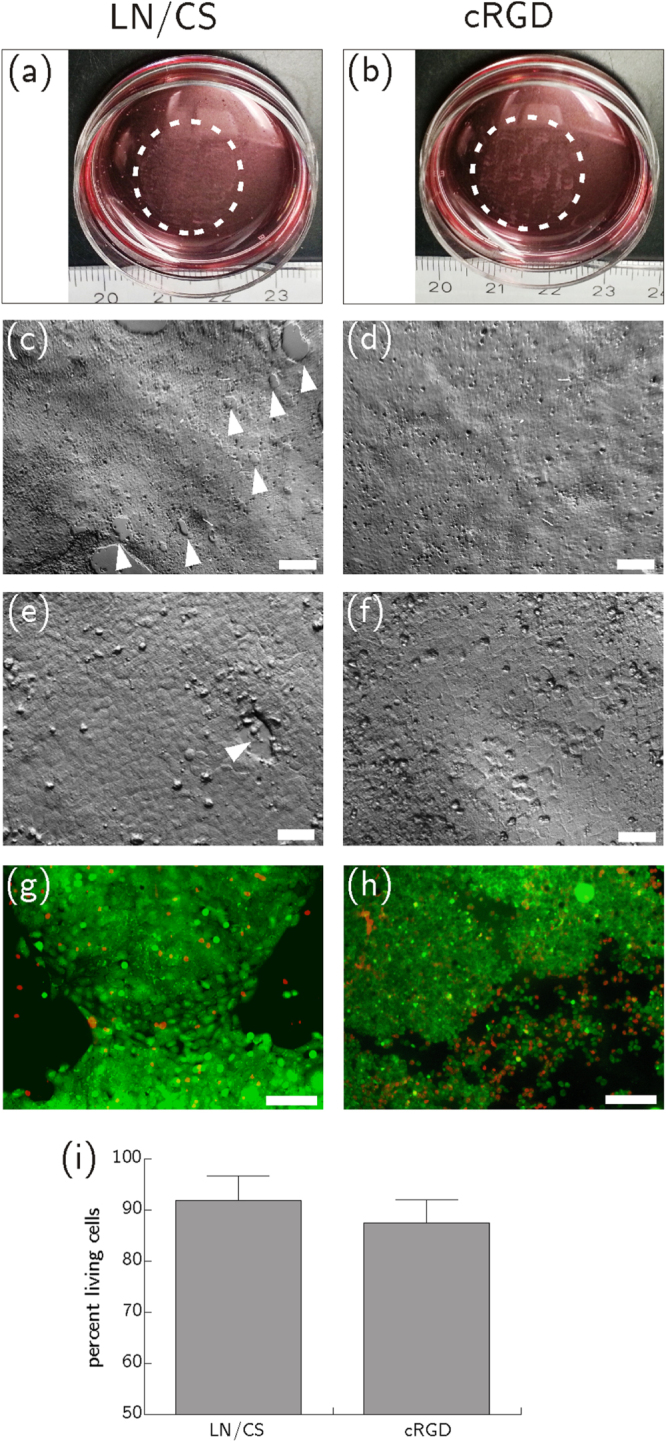
HCEC after thermally induced detachment from PVME_50_–PNiPAAm_40_–PVMEMA_10_ carriers functionalized with LN/CS or cRGD and transfer onto biofunctionalized, polymer-coated cover slips. (a), (b) Macroscopic images show HCEC sheets four hours after the transfer. (c)–(f) Microscopic images of HCEC sheets four hours after the transfer. The few holes that emerged in the fragile cell sheet during detachment and transfer are marked with white arrow heads (scale bar (c) and (d): 200 *μ*m, scale bar (e), (f) 50 *μ*m). (g), (h) Life-dead-staining of HCEC one day after transfer. Vital cells are shown in green (FDA), necrotic cells in red (PI) (scale bar 130 *μ*m). (i) Flow cytometric analysis of HCEC after vital staining with PI. Better cell survival was seen after transfer from LN/CS-functionalized SRP carriers than after transfer from cRGD-functionalized samples. The difference between LN/CS-functionalized and cRGD-functionalized SRP carriers was not statistically significant (*p* = 0.315). Mean ± sd, *n* = 3.

After complete and fast detachment HCEC were successfully transferred as nearly complete sheets onto planar culture substrates using stabilizing cellulose acetate membranes. Only a few holes emerged in the fragile cell sheet during detachment and transfer. These holes closed after transfer and re-attachment to a new surface within three to five days. Typical polygonal morphology and smooth monolayering of cells was retained four hours after transfer (figures 5(a)–(f)), but the following day some areas of the transferred sheets appeared aggregated and irregular. HCEC sheets contained only few dead (mainly necrotic) cells one day after transfer (figure S8). Better cell survival was seen after transfer from LN/CS-functionalized PVME_50_–PNiPAAm_40_–PVMEMA_10_ carriers than after transfer from cRGD-functionalized samples (figures 5(g)–(i) and S8) but the difference between LN/CS-functionalized and cRGD-functionalized SRP carriers was not statistically significant (*p* > 0.05).

### 3.5. Molecular characterization of HCEC after transfer onto planar targets or porcine corneas

**Figure 6.**
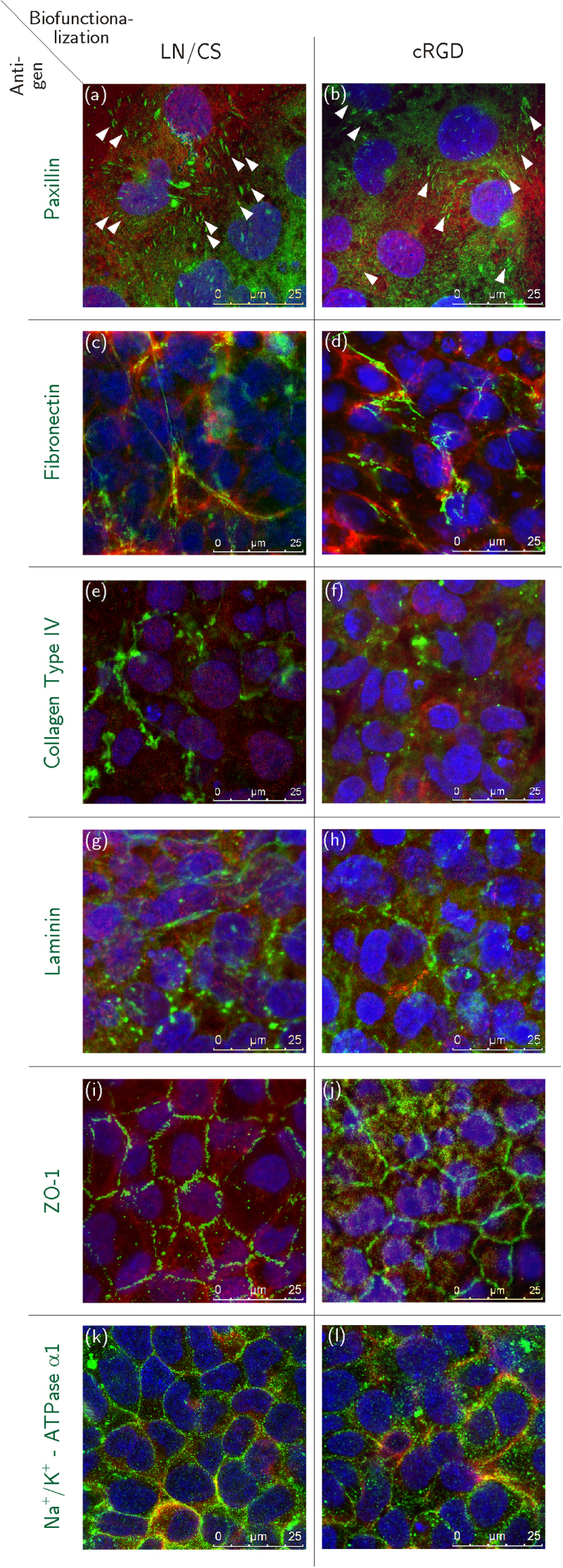
Immunocytochemical staining of HCEC 24 h after detachment and transfer. HCEC were cultured for eight days on PVME_50_–PNiPAAm_40_–PVMEMA_10_ carriers functionalized with LN/CS or cRGD and subsequently transferred onto biofunctionalized, polymer-coated cover slips. (a), (b) Ellipsoid shaped paxillin signals (white arrowheads). (c), (d) Fibronectin fibers. (e), (f) Collagen type IV fibers. (g), (h) Laminin. (i), (j) Correct lateral localization of membrane-bound ZO-1. (k), (l) Na^+^/K^+^-ATPase *α*1 associated to the lateral cell membranes. Antigens of interest are shown in green (Alexa Fluor^®^488), F-actin fibers in red (Phalloidin) and the nuclei in blue (Hoechst). *n* = 3.

Many ellipsoid paxillin signals were observed closely to F-actin fiber termini in all transferred HCEC sheets irrespective of the kind of biofunctionalization during the preceding culture phase (figures 6(a) and (b)). The deposition and morphology of fibronectin and collagen type IV did not change markedly upon detachment and transfer (figures 6(c), (d)–(f)), while a few LN signals appeared as fine fibers after transfer from LN/CS-functionalized samples (figures 6(g) and (h)). The appearance of ZO-1 (figures 6(i) and (j)) remained unchanged after transfer, while Na^+^/K^+^-ATPase *α*1 was more laterally located than before detachment (figures 6(k) and (l)).

**Figure 7.**
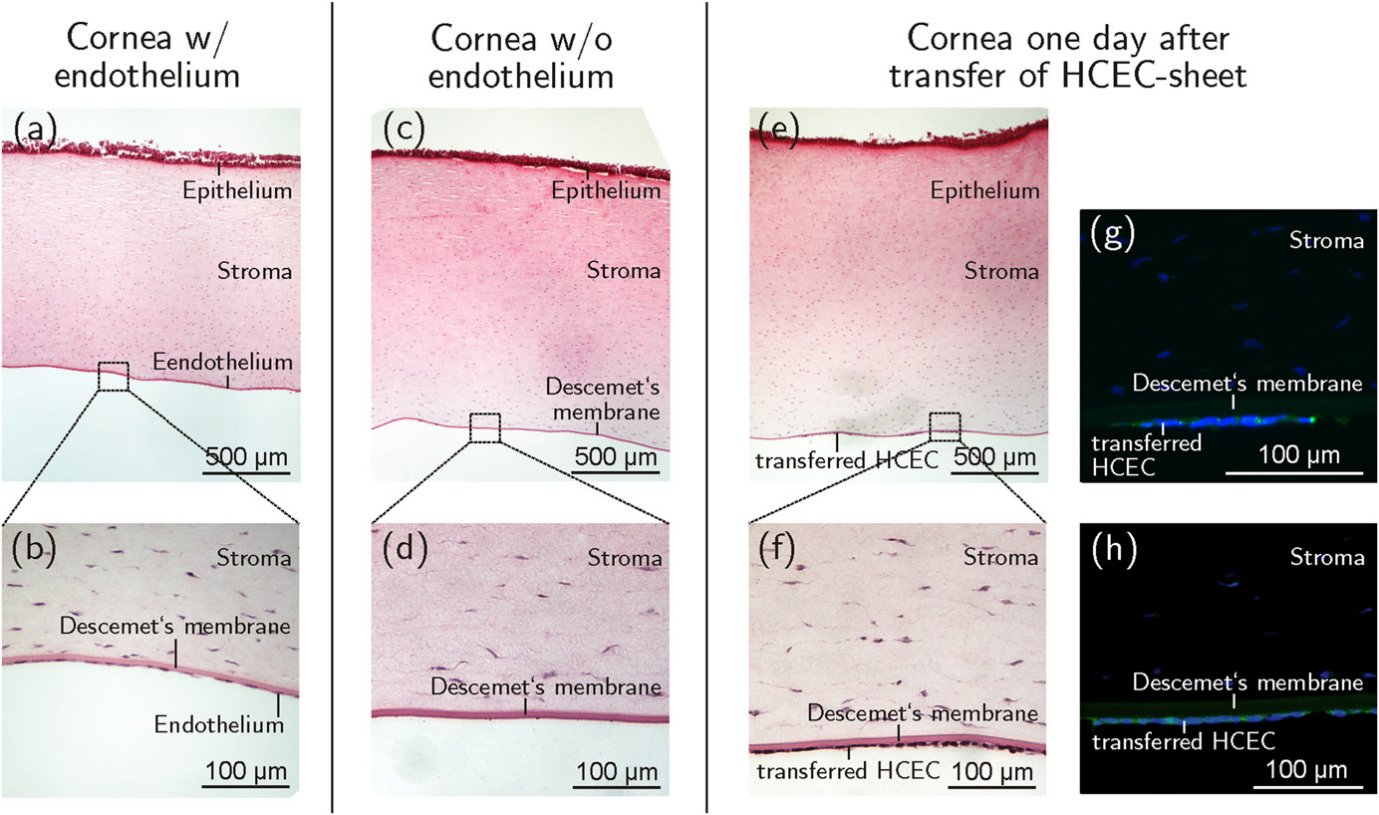
Sagittal sections of porcine corneas. (a), (b) The corneal endothelium can be seen as a monolayer of squamous cells at the posterior corneal side before de-endothelialization. (c), (d) After removal of the porcine endothelium the bare Descemet’s membrane is visible. (e), (f) One day after transfer of HCEC-sheets onto de-endothelialized corneas the cells appeared attached to Descemet’s membrane as a confluent monolayer. Positive immunohistochemical staining against ZO-1 (g) and Na^+^/K^+^-ATPase *α*1 (h) of transferred HCEC-sheets shown in green (Alexa Fluor^®^488), nuclei are displayed in blue (DAPI). *n* = 3.

One day after the transfer of HCEC sheets from PVME_50_–PNiPAAm_40_–PVMEMA_10_-carriers onto de-endothelialized porcine corneas (figures 7(c) and (d)), multilayered and monolayered HCEC sheets could be demonstrated (figures 7(e) and (f)). Immunostaining revealed that the correct lateral, membrane-bound localization of the function-associated proteins ZO-1 (figure 7(g)) and Na^+^/K^+^-ATPase *α*1 (figure 7(h)) was not altered by the transfer process, thereby closely resembling the *in situ*-situation. These results are proof of principle for the successful transfer of HCEC sheets grown and detached from thermo-responsive PVME_50_–PNiPAAm_40_–PVMEMA_10_ carriers to de-endothelialized whole corneas.

## 4. Discussion

The primary aim of this study was to provide an improved thermo-responsive carrier for the stimulated detachment of functional tissue engineered human corneal endothelial transplants. Therefore, a thermo-responsive cell culture carrier with adjustable physico-chemical and biomolecular properties was developed and adapted to the demands of HCEC. Since primary HCEC are rarely available due to respective legislation [30], this carrier was tested using the immortalized cell line HCEC-12 as a model. HCEC-12 has a morphology very similar to primary HCEC, although it has an unlimited regenerative capacity. The application of a cell line allowed for the reproducible and comparable biological evaluation of the developed thermo-responsive cell culture carrier while at same time avoiding donor-specific differences associated with primary HCEC.

The overall switching behavior of the novel PVME_50_–PNiPAAm_40_–PVMEMA_10_ blend is comparable to other PVME-based systems [5], though its curve shape is less distinct. The proposed PVME_50_–PNiPAAm_40_–PVMEMA_10_ blend with its manifold tunable properties represents a versatile system for culture and stimulated detachment of cell sheets. The covalent protein binding mechanism provided by the blend system is the key to an effective functionalization that allows for tuning the balance between initial cell adhesion and stimulated detachment. The approach is suitable for generating transferable HCEC monolayers that maintain their typical morphology. Initially, weaker cell adhesion on PVME_50_–PNiPAAm_40_–PVMEMA_10_ was observed, however the carriers had no negative effect on viability of cultured HCECs. It is known that HCEC interact with the RGD motif of various ECM molecules via *α*v*β*3, *α*v*β*5, and *β*1-integrins [31–38]. Considering that LN contains various binding motifs, like YIGSR and SIKVAV, but only one RGD motif per molecule [37, 39], it becomes clear that cRGD-functionalized samples present more adhesion promoting ligands at their surface than LN/CS-functionalized samples. Accordingly, biofunctionalization with cRGD supported better HCEC adhesion, but was less appropriate for an efficient detachment of HCEC sheets. As a reasonable compromise, the design of peptides which combine the adhesion promoting function of RGD and the proliferation promoting function of YIGSR might further optimize thermo-responsive cell culture carriers [38, 40, 41]. We observed a gradual increase in secreted matrix proteins as seen during the generation of Descemet’s membrane during corneal development [42–45]. Further findings on the localization of functional proteins indicate that morphological fundamentals for the physiological function of cultured HCEC sheets can be achieved on thermo-responsive PVME_50_–PNiPAAm_40_–PVMEMA_10_ carriers. Additional measurements of TEER confirmed that HCEC sheets grown on PVME_50_–PNiPAAm_40_–PVMEMA_10_ exhibited a leaky pump function as described by Fischbarg *et al* for native corneal endothelium [46]. The difference of 1 *Ω* cm^2^ in TEER between samples with or without SRP coating is most likely due to the coating as such, and is not due to any alteration in cellular behavior. In summary, with respect to the results obtained for LN/CS-functionalized samples concerning (i) the formation of morphologically and functionally adequate HCEC layers, (ii) the easier detachment of sheets and (iii) better results for the viability of cells detached from LN/CS-functionalized PVME_50_–PNiPAAm_40_–PVMEMA_10_ carriers, LN/CS-functionalized samples are favored for this particular application.

Detachment is regulated by the swelling behavior, stiffness, and chemical properties of the polymer [47]. The PNiPAAm component shows a low affinity to electron beam cross-linking and may only be loosely integrated into the highly cross-linked PVME network, increasing the flexibility of the polymer network and supporting HCEC sheet detachment. Further extrinsic parameters also influence cell detachment, e.g. complete HCEC detachment was observed after incubation at 4 °C. Similarly, Reed *et al* saw best detachment of bovine aortic endothelial cells after incubation at 4 °C, while Okano *et al* observed efficient cell detachment above 20 °C, from pure PNiPAAm coatings [48, 49]. Since HCEC have thermo-sensitive ion channels responding to cold or heat with an increased Ca^2+^ influx [50–52], cold-induced detachment may influence cell survival due to Ca^2+^-activated signaling. It is known that hypothermic storage of donor corneas at 4 °C, which is usually performed for up to 14 days, can lead to elevated cell death of corneal endothelial cells [53–55], e.g. cell death was induced in cell cultures of corneal endothelium already after 18 h of cold storage [54]. Though it has not been investigated to date if incubating the samples at 4 °C for 60 min only is sufficient to induce cell death, a potentially adverse effect on cell viability cannot be excluded. Our results showed a high viability of HCEC after the detachment and transfer onto new substrates (figures 5 and S8) after being incubated at 4 °C for 1 h. Nevertheless, modifying the SRP properties to enable detachment at temperatures >4 °C may further improve the method. Other factors influencing the optimal detachment temperature are cellular processes like cytoskeletal remodeling, the ECM, or culture medium composition [4, 48].

An imbalance of cellular traction forces during detachment may have been passed on to tight and adherens junctions via the cytoskeleton, impairing junctional integrity and leading to holes in formerly confluent cell sheets. Irregularities in the cell sheet structure after transfer and adhesion may also be a consequence of uncompensated inherent cellular traction forces, though ECM constituents were detached and transferred together with the HCEC sheet, supporting its integrity and aiding adhesion onto the target [56]. After transfer, the production and localization of functional proteins ZO-1 and Na^+^/K^+^-ATPase resembled the *in situ* situation more closely than before. Ide *et al* made a similar observation and concluded that tight junctions were preserved during thermally induced detachment and transfer [43].

The transfer of cell sheets from planar carriers onto a concave target such as the cornea involves adaptation to the corneal curvature. Radial cutting of the cellulose acetate membrane and gentle centrifugation improved the transfer process, but could not satisfactorily solve the problem. Furthermore, the additional mechanical stress from this procedure led to folding of the sheets and impaired their morphology. Stable adhesion of transferred sheets to concave target tissues is a challenge that urgently needs to be addressed if implementation into pre-clinical and clinical settings is to be achieved. Alternative tools such as flexible membranes or gels [57, 58] might optimize the transfer of HCEC sheets onto concave target structures.

## 5. Conclusions

Electron beam cross-linked PVME_50_–PNiPAAm_40_–PVMEMA_10_ provides a broad range of physico-chemical and biomolecular tuning parameters to meet cell type specific requirements for balanced cell adhesion and detachment. This new cell culture carrier was successfully applied to generate and transfer transplantable sheets of HCEC while maintaining appropriate cell morphology, viability and functionality as demonstrated by a comprehensive biological analysis of the cell layers *before and after* detachment and transfer. Cell sheet transplantation onto de-endothelialized porcine corneas was performed *in vitro* as a proof-of-principle experiment towards future clinical application.
